# Biologically Targeted Radiation Therapy: Incorporating Patient-Specific Hypoxia Data Derived from Quantitative Magnetic Resonance Imaging

**DOI:** 10.3390/cancers13194897

**Published:** 2021-09-29

**Authors:** Emily J. Her, Annette Haworth, Yu Sun, Scott Williams, Hayley M. Reynolds, Angel Kennedy, Martin A. Ebert

**Affiliations:** 1 School of Physics, Mathematics and Computing, University of Western Australia, Perth, WA 6009, Australia; Jungmin.Her@health.wa.gov.au (E.J.H.); Martin.Ebert@health.wa.gov.au (M.A.E.); 2 Institute of Medical Physics, University of Sydney, Sydney, NSW 2006, Australia; yu.sun@sydney.edu.au; 3 The Sir Peter MacCallum Department of Oncology, University of Melbourne, Melbourne, VIC 3000, Australia; Scott.Williams@petermac.org; 4 Division of Radiation Oncology, Peter MacCallum Cancer Centre, Melbourne, VIC 3000, Australia; 5 Auckland Bioengineering Institute, University of Auckland, Auckland 1010, New Zealand; hayley.reynolds@auckland.ac.nz; 6 Department of Radiation Oncology, Sir Charles Gairdner Hospital, Perth, WA 6009, Australia; angel.Kennedy@health.wa.gov.au; 7 5D Clinics, Perth, WA 6010, Australia

**Keywords:** prostate cancer, hypoxia, tumour control probability, radiogenomics, multiparametric MRI

## Abstract

**Simple Summary:**

Recent clinical trials have demonstrated the capability of safely delivering prostate radiotherapy with a simultaneous focal boost. These studies have also indicated limitations to achieving focal boosts whilst trying to limit normal tissue toxicity. More guidance in the location and level of the required boost could alleviate such limitations. Tumour hypoxia is one of the causes of clinically observed radioresistance and hypoxic volumes represent prime candidates for a focal boost. Multiparametric magnetic resonance imaging (mpMRI) has the potential to quantitatively describe the extent and spatial distribution of hypoxia in prostate cancer. In this article we demonstrate a biologically targeted radiotherapy approach that can utilise this information to target hypoxia for favourable treatment outcomes.

**Abstract:**

Purpose: Hypoxia has been linked to radioresistance. Strategies to safely dose escalate dominant intraprostatic lesions have shown promising results, but further dose escalation to overcome the effects of hypoxia require a novel approach to constrain the dose in normal tissue.to safe levels. In this study, we demonstrate a biologically targeted radiotherapy (BiRT) approach that can utilise multiparametric magnetic resonance imaging (mpMRI) to target hypoxia for favourable treatment outcomes. Methods: mpMRI-derived tumour biology maps, developed via a radiogenomics study, were used to generate individualised, hypoxia-targeting prostate IMRT plans using an ultra- hypofractionation schedule. The spatial distribution of mpMRI textural features associated with hypoxia-related genetic profiles was used as a surrogate of tumour hypoxia. The effectiveness of the proposed approach was assessed by quantifying the potential benefit of a general focal boost approach on tumour control probability, and also by comparing the dose to organs at risk (OARs) with hypoxia-guided focal dose escalation (DE) plans generated for five patients. Results: Applying an appropriately guided focal boost can greatly mitigate the impact of hypoxia. Statistically significant reductions in rectal and bladder dose were observed for hypoxia-targeting, biologically optimised plans compared to isoeffective focal DE plans. Conclusion: Results of this study suggest the use of mpMRI for voxel-level targeting of hypoxia, along with biological optimisation, can provide a mechanism for guiding focal DE that is considerably more efficient than application of a general, dose-based optimisation, focal boost.

## 1. Introduction

Hypoxia has been widely reported to exist in prostate cancer (PCa) and has been shown to be linked to local treatment failure, increased risk of metastases and radioresistance [1]. To overcome the effects of radioresistance, radiation doses in excess of 20% above standard prescription doses have been proposed [2]. Conventional radiotherapy treatments for PCa aim to deliver a uniform dose of radiation to the entire prostate, and further dose escalation (DE) beyond standardly prescribed doses is limited due to the increased risk of toxicity. The recently reported FLAME study demonstrated that, by increasing the dose only to the tumour visible on multiparametric magnetic resonance imaging (mpMRI), improvements in tumour control could be achieved when compared with whole gland treatments without a boost dose to the tumour [3]. The median boost dose delivered to the tumour in this trial was only 10% higher than the whole gland dose and therefore likely insufficient to overcome hypoxic sub-volumes. To further increase the boost dose to the tumour (without increasing toxicity), the study investigators conducted a similar Phase II trial with a hypofractionated schedule, called the hypo-FLAME study [4]. PCa is known to be sensitive to the fractionation schedule, that is, increasing the dose delivered at each treatment and reducing the number of treatments (fractions) is more effective than a low dose/fraction schedule [5]. The hypo-FLAME study demonstrated that delivering the treatment in five treatment fractions rather than thirty-five (the number of fractions used in the FLAME study) achieved acceptable rates of acute toxicity. However, the median dose delivered to the boost volume (40.3 Gy) was less than the intended dose of 50 Gy due to the strict requirement to adhere to the specified organs at risk (OAR) dose constraints. Hence, a new strategy to overcome radioresistant tumours is required.

Conventional dose-based planning approaches aim to deliver a prescribed dose to the prostate (and a boost dose to the tumour if applicable). In contrast, biologically targeted radiation therapy (RT) considers the specific characteristics, on a voxel-wise basis, of the tumour in the treatment planning process, and aims to maximise the tumour control probability of each voxel whilst minimising normal tissue complication probability. Biologically targeted RT has, up until recent times, not been widely practised due to the inability to spatially map tumour characteristics. Quantitative imaging, however, has demonstrated an ability to define pathological and physiological features of tissue at the voxel-level and hence the potential to provide patient-level input parameters for a biological approach to treatment planning [6]. In the review article of Sun et al. for example, it was demonstrated that machine learning methods can be used to define spatial maps of tumour cell density and regions of high and low grade disease [7]. These studies typically correlated features in mpMRI with ground-truth histology using sophisticated image co-registration frameworks such as those defined by Reynolds et al. [8,9]. Using these methods to define tumour hypoxic sub-volumes however, has, up until recently, proven to be challenging as hypoxic regions cannot be directly defined on histology and used to correlate with imaging features. To address this issue, Sun et al. used a radiogenomics approach to provide a surrogate for ground-truth hypoxia for correlation with imaging features [10]. This study identified sixteen candidate features in mpMRI that correlated with the hypoxic signatures expressed in surgical specimens of prostate cancer.

In this article, we present the first in silico study to investigate the benefits of a biologically targeted RT approach (which we define as “BiRT”) to overcome the effects of hypoxic tumour sub-volumes. We compare this approach with dose-based optimisation methods using the hypo-FLAME dose fractionation schedule, and include whole-gland treatments, whole-gland treatments with a tumour-defined boost volume, and whole-gland treatments with a boost volume and hypoxic sub-volume.

## 2. Materials and Methods

### 2.1. Tumour Biology Maps

**Figure 1:**
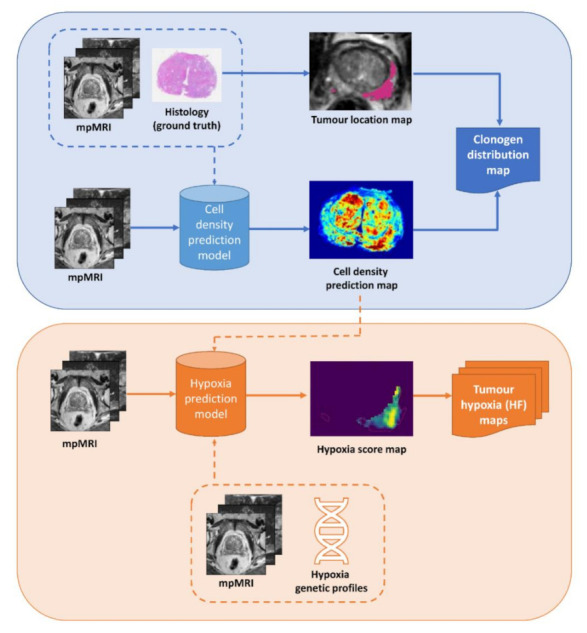
Schematic diagram showing the process for creating the clonogen distribution map (upper panel, scaling not shown) and the tumour hypoxia maps (lower panel).

All radiotherapy treatment plans utilised in vivo mpMRI data from five participants scheduled for radical prostatectomy as part of a Human Research Ethics Committee approved project (Reference number: HREC/15/PMCC125) [9]. Patient demographics are summarised in Table 1. Patient-specific cell density prediction maps were derived for each patient from mpMRI using predictive models developed by Sun et al. [11]. The tumour volume was defined using co-registered ground-truth histology data according to the methods of Reynolds et al. [9]. The clonogen distribution maps were computed for each patient by multiplying the binary tumour location map and cell density prediction map. Voxels defined as non-tumour (i.e., with a value of 0 in the tumour location map) were assigned a clonogen density of 0. Otherwise, the cell density value was retained as a relative measure of clonogen density. As the cell density prediction map did not differentiate clonogens from normal cells, the clonogen distribution maps were linearly scaled to acquire a median total clonogen number of 10^7^ for the five patients (Table 2), which is the estimated population-median total clonogen number of high-risk PCa [12]. The scaled clonogen distribution map was used in the calculation of tumour control probability (TCP) as shown in Figure 1.

Patient-specific hypoxia score maps were derived from mpMRI using the top eight performing candidate radiomics features identified by Sun et al. [10]. Briefly, these candidate features were derived from a radiogenomics study that investigated the relationship between PCa tissue expressing genetic profiles shown to be associated with hypoxia, and radiomics features extracted from the co-registered in vivo mpMRI data [9]. The study described by Sun et al. [10] provided a binary output representing hypoxic or normoxic status. To constrain the model to search only within tumour-bearing regions likely to contain hypoxia, we assumed that oxygen consumption is a function of cell density [13]. Hence, the hypoxia score map was derived from a weighted sum of the cell density and the selected eight texture features (Figure 1). Four different hypoxic fractions (HF, percentage of hypoxic voxels within the tumour) were simulated: 20%, 40%, 60% and 80%. For each patient, binary tumour hypoxia maps of each of the four HFs were generated by using thresholds that equate to the 80th, 60th, 40th and 20th percentiles of the hypoxia scores of the tumour voxels, respectively.

The voxel resolution of the tumour biology maps was resampled to 2 mm × 2 mm × 2.5 mm from the original resolution of 0.22 mm × 0.22 mm × 2.5 mm for improved computation time.

As patient data did not include CT imaging, a single CT image set (used for all patients) was selected from an established clinical trial for dose calculation purposes. The selected CT data set contained a prostate volume that was similar in shape and size to the MRI defined prostate volumes of the five patients, but slightly larger such that the prostate contours delineated on the T2w MRI were wholly contained within the CT-defined prostate contour. The organs at risk (OARs) including the rectum, bladder, and head of femurs (HOFs) were delineated on the CT images.

### 2.2. TCP Model

All model equations are presented in Table 3 and associated parameters defined in Table 4. The TCP of each treatment plan was calculated using a revised radiobiological model of Haworth et al. [14]. The TCP of the i^th^ voxel of the clinical target volume (CTV) with N voxels for fractionated external beam radiotherapy consisting of *n* fractions was calculated using Equation [1]. In this implementation, the radiosensitivity parameter α was assumed to be log-normally distributed within a population and the population-weighted TCP for the i^th^ voxel is given by Equation [2]. Most model parameters were derived from the work of Wang et al. [12]. Tumour characteristics were assumed to remain constant during treatment. For a CTV voxel classified as hypoxic, the α and α/β parameters were scaled by the clinical oxygen enhancement ratio (OER) as in Equations [3] and [4]. The nominal value of OER used in this study was 1.4, with values of 1.2 and 1.6 considered in a sensitivity analysis [15]. Assuming that individual voxel responses are independent, the overall TCP is given by Equation [5].

### 2.3. Treatment Planning

Treatment planning was performed using a MATLAB based open-source program, matRad (German Cancer Research Centre, Heidelberg, Germany, version 1.4 beta, [22,23]. matRad simulates 6 MV linear accelerator beams using pre-calculated beamlet kernels for user-defined beam angles [22,23]. Beamlet weightings are optimised by a gradient descent algorithm (version 1.4 beta) incorporating direct aperture optimisation. The original code was modified to include the biological optimisation functions and voxel-level model parameters. matRad was executed using MATLAB (version 2018b, The MathWorks Inc., Massachusetts, USA). The beamlet width was 2.5 mm, and a 7-field beam geometry (0°, 40°, 80°, 110°, 250°, 280°, 310°) was used. A beamlet width of 2.5mm was chosen to match the worst CT resolution in the z axis.

**Figure 2:**
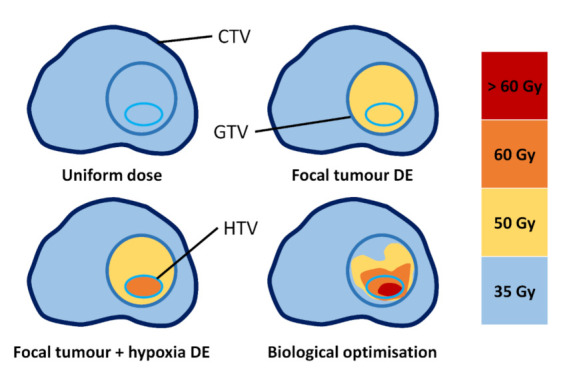
The four different planning approaches used in this study. The uniform dose method aimed to produce a treatment plan that would achieve 35 Gy delivered to the entire prostate. The Focal DE approach aimed to deliver 35 Gy to the entire prostate with a boost dose of 50 Gy to the tumour. The focal tumour + hypoxia DE plan aimed to deliver 35 Gy to the entire prostate, 50 Gy to the tumour and 60 Gy to the hypoxic sub-volume. Biologically optimised plans aimed to maximise the tumour control probability whilst minimising the normal tissue complication probability.

Four treatment planning methods were explored in this study: (1) uniform-dose planning, (2) focal DE to the tumour, (3) focal DE to the tumour and hypoxic sub-volumes and (4) robust biological optimisation. Each of these methods are presented in schematic format in Figure 2. The first three are classified as dose-based planning methods. Uniform-dose planning and tumour DE methods were included as non-hypoxia-targeting planning methods for comparison with the hypoxia targeting methods. The dose prescriptions and dose-volume (DV) constraints for the dose-based planning methods were based on the hypo-FLAME trial protocol where patients were treated with ultra-hypofractionated treatment (35 Gy/5 fractions) to the whole prostate gland with a focal boost up to 50 Gy to the mpMRI-defined tumours [4]. Detailed planning constraints for all planning methods are summarised in Table 5.

#### 2.3.1. Method 1: Uniform-Dose Planning

The main objective of the uniform-dose planning method was to provide a homogeneous dose prescription of 35 Gy to the planning target volume (PTV) while satisfying OAR DV constraints. The hypo-FLAME trial recommended a planning margin of 4-5 mm, however, for consistency across all 4 planning approaches we accounted for geometric uncertainties using a modelling approach to produce the robust biologically optimised treatment plans (Method 4). These uncertainties were considered by noting that random uncertainties increasingly behave like systematic errors with hypofractionation [24]; the geometric uncertainty was modelled as consisting of a purely systematic component [25,26,27] with an effective systematic error, $\Sigma^{\prime }$.

The overall distribution and the resultant margin for the three principal directions used for uniform-dose planning are summarised in Table 6 and are based on the geometric uncertainties from target delineation, fiducial marker localisation and intrafraction prostate motion [19,20,21].

The OAR dose constraints were identical to those used for the hypo-FLAME trial. The PTV and CTV treatment planning objectives were V33.25Gy (volume receiving 33.25 Gy, 95% of the prescription dose) ≥ 99% and V35Gy (volume receiving 35 Gy, 100% prescription dose) ≥ 99%, respectively (Table 5).

#### 2.3.2. Method 2: Focal Tumour DE

The tumour voxels were segmented and defined as the gross tumour volume (GTV). Based on the hypo-FLAME trial objectives, the focal DE plans were designed to deliver a dose of 35 Gy to the PTV and up to 50 Gy to the GTV while satisfying the OAR dose constraints.

#### 2.3.3. Method 3: Focal Tumour + Hypoxia DE

The voxels classified as hypoxic were segmented as hypoxic target volumes (HTVs) for each HF. A previous Monte Carlo study estimated that an increase of 20–50% of tumour dose is required to increase the TCP if a significant portion of chronic hypoxia is identified [2]. As such, a DE up to 60 Gy to the HTV was considered while satisfying the OAR dose constraints.

#### 2.3.4. Method 4: Biologically Targeted Radiotherapy Approach

Uncertainties in the delivery of highly modulated treatment fields can result in significant differences between planned and delivered dose distributions. Hence, incorporating robust treatment planning approaches is necessary for biologically targeted radiotherapy approaches. In this study we utilise the term “expectation value” to indicate the reported values of TCP and normal tissue complication probability (NTCP) account for treatment delivery uncertainties and are termed <TCP> and <NTCP> respectively. In addition to the uncertainties included in the planning margin for the uniform-dose planning approach, uncertainties associated with each of the image-registration steps were included for biological optimisation. These were MR-to-histology registration [9] and MR-to-CT registration uncertainties [28,29,30]. They were assumed to be isotropically distributed about a mean of 0 mm. Uncertainties in the models to predict tumour location and cell density have been previously quantified [11,31]. The TCP model demonstrated low sensitivity in these parameters [32], therefore, the uncertainties in predictive modelling were not considered. The overall distribution in three principal directions used for biological optimisation is given in Table 6.

Biological optimisation aimed to maximise <TCP>, while simultaneously minimising <NTCP>, of the rectum and bladder. The methods described in Witte et al. [33] were used to modify the biological objective functions to calculate <TCP> and <NTCP>. The impact of effective systematic error was simulated by the translation of the patient body relative to the dose distribution. Rotational uncertainties were not considered, and shift-invariance of the dose distribution was assumed. Equations [1], [2] and [5] were modified to form Equations [6] and [7].

NTCP is based on the generalised equivalent uniform dose (EUD) [34]. The expectation value of EUD in 2 Gy fractions, <EUD_2Gy_>, was computed using Equation [8], and <NTCP> was then calculated via Equation [10].

### 2.4. Aim 1: Effect of Hypoxia on <TCP> of Dose-Based Planning Methods

**Figure 3:**
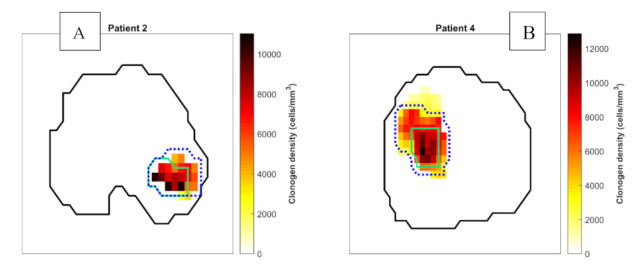
Axial images showing hypoxic sub-volumes within the prostate (black) for patient 2 (**A**) and patient 4 (**B**) representing the smallest and largest tumour volumes of the study cohort respectively. The volumes containing 20% and 80% of hypoxic voxels are represented by green solid lines and blue dotted lines, respectively. Note this is a 2-dimensional representation of a 3D volume.

To investigate the effect of hypoxia on <TCP> of dose-based planning methods, <TCP> of the plans generated with uniform-dose (Method 1), focal tumour DE (Method 2) and focal tumour+hypoxia DE (Method 3) approaches were calculated for all oxygenation status considered: HF of 0% (normoxic), 20%, 40%, 60% and 80% (see Figure 3).

### 2.5. Aim 2: Comparison of Normal Tissue Effects in Focal DE and Biologically Optimised Plans for Targeting Tumour Hypoxia

The overall dose distributions of the biologically optimised plans (Method 4) were linearly scaled to attain the same <TCP> as the corresponding focal tumour + hypoxia DE plans (Method 3). Biologically optimised plans were assumed to be invariant with dose scaling. Dose to the rectum and bladder was compared by calculating the <EUD>. Mean doses were evaluated for the HOFs.

A paired *t*-test was performed to compare focal tumour + hypoxia DE plans and biologically optimised plans of the same HF.

### 2.6. Sensitivity to Oxygen Enhancement Ratio (OER)

To investigate the sensitivity of robust biological optimisation to different OER values, hypoxia-targeting biological plans were generated for patients with the lowest and the highest total number of clonogens (Patient 2 and 4) and modelling of the HF at 20% and 80% (Figure 3). Including the nominal OER of 1.4 from Wang et al.’s study, the lower and upper 95% confidence interval values of 1.2 and 1.8 were investigated [15]. The sensitivity was measured by variations in dose to the CTV, rectum and the bladder.

### 2.7. Statistical Analysis

All statistical analyses were performed with the R statistical language (R Foundation for Statistical Computing, Austria, Version 3.4.2). A test statistic (*p*-value) less than 0.05 was considered significant.

## 3. Results

All treatment plans were generated with dose to OARs within the specified dose constraints (shown in Table 5). Method 2 treatment plans (focal tumour DE) were designed to deliver up to 50 Gy to the tumour volume. This goal was met in only one treatment plan, with a median dose of 44.7 Gy for the five patients. This is comparable to the median dose of 40.3 Gy reported in the hypo-FLAME study [4]. For Method 3 (focal tumour + hypoxia DE) the mean dose delivered to the hypoxic target volumes ranged from 57.3 Gy –59.7 Gy across all five patients.

### 3.1. Effect of Hypoxia on <TCP> of Dose-Based Planning Methods

**Figure 4:**
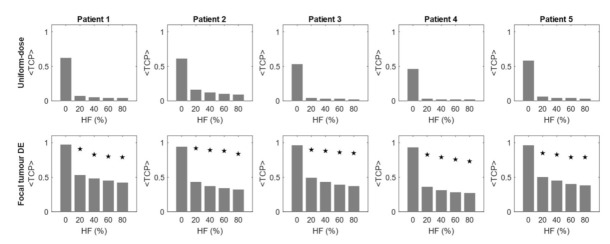
<TCP> calculated for different planning strategies when evaluated with varying hypoxic fractions (HF). (**Top row**): Uniform-dose plans (Method 1). (**Bottom Row**): Focal tumour dose escalation (DE) plans (Method 2). Stars indicate the <TCP> of focal tumour + hypoxia DE plans (Method 3).

The first aim of our study was to compare the <TCP> calculated for treatment planning Methods 1–3 for each of the HF. These results are summarised in Figure 4. Uniform-dose plans (Method 1) resulted in an average <TCP> of 0.56 under normoxic conditions. Focal tumour DE (Method 2) plans demonstrated a significant improvement in normoxic <TCP>, with an average <TCP> of 0.95. However, with the introduction of hypoxia, both planning methods demonstrated a considerable reduction in <TCP>. When plans were re-optimised to incorporate a focal DE of 60 Gy to the HTV (Method 3) a considerable increase in <TCP> was found.

### 3.2. Comparison of Focal DE and Biologically Optimised Plans for Targeting Tumour Hypoxia

**Figure 5:**
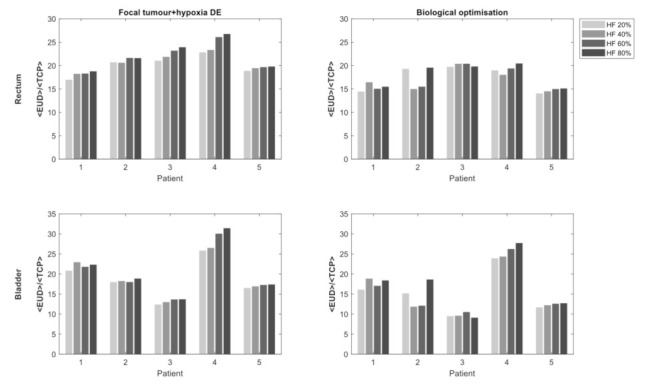
Comparison of rectal and bladder <EUD> with a varying hypoxic fraction (HF). Rectal and bladder <EUD> are normalised to the <TCP> of the focal tumour + hypoxia dose escalation (DE) plans.

Figure 5 shows the rectal and bladder <EUD> of the isoeffective (normalised by <TCP>) focal tumour+hypoxia DE (Method 3) and biologically optimised plans (Method 4). As expected, the sparing of the rectum and bladder became increasingly difficult with increasing HF, seen in both hypoxia-targeting planning methods. Biologically optimised plans displayed slightly varying response to increasing HF, potentially due to the size and location of the tumour, as well as the location of the hypoxic region within the tumour, especially at lower HF. For all HFs considered, biologically optimised plans could achieve statistically significant lower rectal and bladder <EUD> compared to isoeffective focal tumour + hypoxia DE plans for the entire patient cohort (*p*-values < 0.01). Differences in mean dose to HOFs were not statistically significant.

### 3.3. Sensitivity of Robust Biological Optimisation to OER

**Figure 6:**
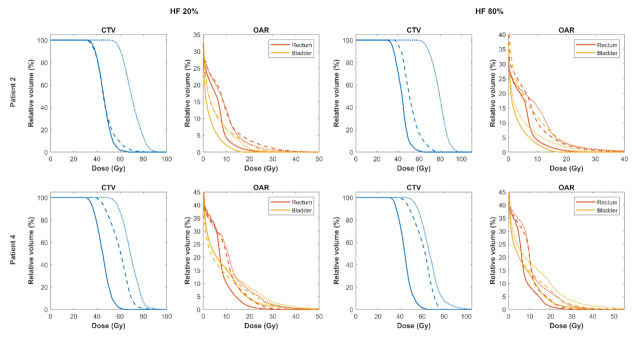
DVH comparison of isoeffective biologically optimised plans with varying oxygen enhancement ratio (OER) (solid line = 1.2, dashed line = 1.4, dotted line = 1.8).

The results so far applied an OER value of 1.4 as our radiogenomics study was not designed to provide a quantitative measure of OER. As shown by Figure 6, biological optimisation (Method 4) demonstrated a high sensitivity to the OER value as anticipated, since the TCP model is most sensitive to radiosensitivity parameters [32]. In the two cases examined, rectal and bladder <EUD> increased with OER along with the mean tumour dose for both HFs considered. Bladder <EUD> for Patient 2 decreased slightly from OER of 1.4 to 1.8. This is likely due to the location of the hypoxic sub-volume in the posterior portion of the prostate close to the rectum. The compromise was made in the bladder, which had a lower weighting than the rectum.

## 4. Discussion

Dose escalation (DE) to the GTV using the hypo-FLAME protocol (Method 2) demonstrated a substantial improvement in <TCP> compared to uniform-dose planning (Method 1) planned without consideration of oxygenation and is consistent with higher EQD_2Gy_ doses reported in the FLAME and hypo-FLAME studies (106.3 Gy and 133.3 Gy respectively) [3,4]. In this study, we investigated the effect of introducing varying proportions of hypoxic volumes within the tumour and demonstrated that even with only 20% of voxels affected, there is a significant impact on TCP. Increasing the hypoxic volume reduced the TCP to a smaller degree, suggesting that it is less important to precisely define affected voxels compared with a binary tumour classification of hypoxic versus normoxic. Hypoxia is considered one of the possible reasons for treatment failure after radiotherapy [1] and this study suggests that 50 Gy delivered in an ultra-hypofractionated schedule may be insufficient to overcome the effects of hypoxia. As a relatively high cumulative acute grade 2 genitourinary toxicity of 34% at 90 days post-treatment was reported for the hypo-FLAME [4], further dose escalation to the entire tumour volume using standard treatment planning methods may not be possible. To assess the tissue-sparing effect of the hypoxia-targeting BiRT approach [6], a hypoxia DE protocol (Method 3) was devised under the assumption that the same hypoxia information was available. To the authors’ knowledge, there is no published data on the level of DE required to combat hypoxia in ultra-hypofractionated treatment of PCa. Therefore, we adopted a hypoxia DE dose of 60 Gy, 120% of the tumour boost of 50 Gy. This factor was derived from the work of Popple et al., which is commonly used in hypoxia-targeting DE studies for conventional fractionation [2]. Popple et al. estimated that an increase of 20–50% of tumour dose is required for targeting a significant portion of chronic hypoxia. An additional boost to the HTV was feasible without violation of OAR DV constraints. A considerable improvement in <TCP> was observed compared to boosting the GTV alone (Figure 4). The <TCP> remained high for all HFs simulated, however, the tissue-sparing effect decreased with increasing HF. Nevertheless, statistically significant improvements in rectal and bladder dose compared with the dose-optimised plans (Method 4) was achievable by utilising the patient-specific, hypoxia-targeting BiRT approach (Figure 5).

In this in silico study we applied the dose fractionation schedule of the hypo-FLAME study based on the promising results of this clinical trial. These clinical findings also support the conclusions of our previous work where we demonstrated the normal tissue sparing benefits of biologically targeted, hypofractionated schedules compared with conventional fractionation schedules [35].

In this study we applied the CT scan of a single patient for the purpose of dose calculation. We would expect that the conclusions of our study would be unchanged if we had used a synthetic CT derived from the MR data for each patient due to the relative homogeneity of tissue densities within the prostate and surrounding tissue. Our small sample size of five patients included prostates with a range of cell density distributions and tumour sizes to demonstrate their effects on biological optimisation. The volumes of these prostates ranged from 27.6–57.0 cm^3^ representing typical prostate volumes. Whilst large prostate volumes may impact on the ability to limit the dose to the OARs, we would expect that tumour volume would be the predominant factor in challenging the biologically targeted RT approach we have described. We would propose future studies consider a larger sample size with a range of factors, such as prostate and tumour volume that may determine the upper limit for our proposed approach to be feasible. Furthermore the urethra was not included as an OAR as it could not be easily delineated on the MR datasets. Future studies should include a urethra-sparing approach using contrast or modelling from ground-truth histology for urethra position. As the urethra runs through the centre of the prostate, it is however anticipated that hypoxia-targeting, biologically optimised plans will suffer significantly from “streakiness” in the effort to provide a doughnut-shaped dose distribution. As such, hypoxia-targeting biologically targeted radiotherapy approaches may be better realised with brachytherapy. Biological optimisation of radioactive seed placement for low-dose-rate brachytherapy has previously demonstrated improved tumour control whilst simultaneously minimising dose to OARs, including the urethra when population-based tumour biology information was employed [36]. The results in this study support further investigation of biologically targeted brachytherapy with patient-specific tumour information, though potentially with high-dose-rate brachytherapy due to the low α/β of prostate cancer [5]. An alternative approach would be the use of charged particles, with their high linear energy transfer offering the potential of a reduced OER [37,38]. With a high relative biological effect (RBE), charged particle therapy may provide superior hypoxia-targeting plans and should be kept in mind for future prospective clinical trials.

Limitations in the modelling of hypoxia in this work are acknowledged. Although it is well-known that hypoxia exists in a spatially and temporally heterogeneous distribution, tumour hypoxia is a complex process that is still not well understood. In the absence of ground-truth information, a predictive model of tumour hypoxia could not be developed. Instead, surrogates based on genetic profiling and immunohistochemistry staining of histology were used to derive the candidate features for defining the spatial distribution of hypoxia [10]. Additionally, single values of OER were assumed when most likely a range of values are likely to be present. Whilst future studies aim to further our understanding of the relationship of imaging features and hypoxia, we would propose that clinical translation of our work would incorporate measures of hypoxia expression within biopsy specimens as part of the validation studies. With concerns over the adverse effect of hypoxia on hypofractionation due to the insufficient time for reoxygenation to take place [39], reoxygenation kinetics were not included in our modelling. While many sophisticated TCP models that approximate tumour hypoxia dynamics exist in the literature [40,41,42,43,44], they suffer from large uncertainties. The literature on the types of hypoxia (acute or chronic) and their relative impact on PCa radioresistance is also inconsistent [45,46,47,48,49,50,51,52]. Adaptive therapy would, therefore, be more clinically relevant for patient-specific planning [53]. Ideally, hypoxic prediction maps would be produced at several time points before and during treatment to establish spatio-temporal stability of hypoxia and represented by a continuous variation in OER. MR guided radiotherapy using an MRlinac would provide an ideal opportunity to incorporate such a biologically guided adaptive approach. Work is currently underway to determine optimal mpMRI imaging time points for predicting treatment response (ANZCTR UTN U1111-1221-9589). Other tumour characteristics that were assumed to be constant during treatment, such as the distribution of clonogen density, should also be considered for adaptive therapy approaches.

In addition to hypoxia modelling, the TCP model used in this study has further limitations in describing tumour radiation response. In this study we have chosen to use our previously validated TCP model [36], a model that we have used in a number of subsequent biologically targeted radiotherapy studies [6,35,54]. As in our previous studies, cell-to-cell communication, intrafraction repair and accelerated repopulation were excluded. Cell-to-cell communication may additionally impact on radioresistance [55], and as our understanding of this effect grows, we would suggest that our model could be extended to incorporate this effect. Furthermore, in the absence of reliable information, it was assumed that the elimination of all clonogenic cells was required for tumour control. Thus, the <TCP> calculated in this study do not represent the absolute probability of tumour control but instead were used as a relative indicator of tumour control when comparing two plans. This study was intended as an in silico planning study and designed to demonstrate the potential benefit of a hypoxia-targeted biologically optimised approach to treatment planning, a model that could be extended to consider any tumour types that exhibit extreme hypoxia and any factor that may influence radioresistance.

## 5. Conclusions

This work explored the focal DE and biologically targeted radiotherapy approaches for targeting hypoxia in an ultra-hypofractionated prostate radiotherapy schedule. By using patient-specific, mpMRI-derived cell density and hypoxia maps, improved rectal and bladder sparing was achievable for biological optimisation when compared to isoeffective focal DE plans. The results of this study support further investigation of biologically targeted approaches using intensity modulated radiotherapy, brachytherapy and potentially particle therapy to take advantage of the knowledge of the spatial distribution of tumour heterogeneity.

## Figures and Tables

**Table 1:** Patient demographics.

Patient	Age (Years)	Prostate Volume (cm^3^)	PSA (ng/mL)	Gleason Score of the Dominant Nodule	Pathological StagSe
1	58	28.9	9	9 (5 + 4)	pT3b N0
2	64	30.6	6.1	7 (4 + 3)	pT3a
3	68	28.1	11	7 (4 + 3)	pT3b N1
4	68	57.0	42	7 (4 + 3)	pT3a
5	72	27.6	2.2	7 (4 + 3)	pT3a

**Table 2:** The original and scaled total cell number (clonogen) for each patient.

Patient	Total Cell Number in CTV	
	Original (Normal + Clonogen)	Scaled (Clonogen)
1	2.07 × 10^8^	7.27 × 10^6^
2	1.65 × 10^8^	5.79 × 10^6^
3	4.46 × 10^8^	1.57 × 10^7^
4	1.14 × 10^9^	4.00 × 10^7^
5	2.85 × 10^8^	1.00 × 10^7^

**Table 3:** Equations used for modelling tumour and OAR response. Parameters are defined in Table 4.

Tumour Control Probability		
TCP of voxel i for α sample k	${TCP}_{i}\left(\alpha_{k}\right)=exp\left[-\rho_{i}V_{i}exp\left(-\alpha_{k}{nd}_{i}-\frac{\alpha_{k}{nd}_{i}^{2}}{\frac{\alpha }{\beta }}+ln\left(2\right)\frac{T_{exp}}{T_{pot}}\right)\right]$	[1]
TCP of voxel i over population α distribution	${TCP}_{i}=\overset{M}{\underset{k=1}{\sum }}w\left(\alpha_{k}\right){TCP}_{i}\left(\alpha_{k}\right)$	[2]
α sample k for hypoxic tissue	$\alpha_{k,hypoxic}=\frac{\alpha_{k}}{OER}$	[3]
$\frac{\alpha }{\beta }$ ratio for hypoxic tissue	${\left(\frac{\alpha }{\beta }\right)}_{hypoxic}=\frac{\alpha }{\beta }\times \;OER$	[4]
TCP across whole target volume	$TCP\;=\overset{N}{\underset{i=1}{\prod }}{TCP}_{i}$	[5]
Expectation value of target TCP in presence of geometric uncertainty	$\langle TCP\rangle =\overset{M}{\underset{k=1}{\sum }}w\left(\alpha_{k}\right)\langle TCP\left(\alpha_{k}\right)\rangle$	[6]
Expectation value of target TCP for α sample k in presence of geometric uncertainty	$\langle TCP\left(\alpha_{k}\right)\rangle =\;\underset{j}{\sum }G_{\Sigma^{\prime },j}\overset{N}{\underset{i=1}{\prod }}TCP_{i}\left(\rho_{i,j},\alpha_{k},d_{i}\right)$	[7]
Normal Tissue Response		
Expectation value of EUD in presence of geometric uncertainty	$\langle EUD_{2Gy}\rangle =\underset{j}{\sum }G_{\Sigma^{\prime },j\;}{\left[\frac{1}{N}\overset{N}{\underset{i}{\sum }}{\left(EQD_{2Gy}{}_{i,j}\right)}_{}^{a}\right]}^{\frac{1}{a}}\;$	[8]
Equivalent dose in 2 Gy fractions for voxel i	$EQD_{2Gy,\;i}=d_{i}\times n\times \frac{\frac{\alpha }{\beta }+d_{i}}{\frac{\alpha }{\beta }+2}$	[9]
Expectation value of NTCP in presence of geometric uncertainty	$\langle NTCP\rangle =\frac{1}{1+{\left(\frac{TD50}{\langle EUD_{2Gy}\rangle }\right)}^{x}}$	[10]
NTCP model parameter x	$x=\frac{4}{m\sqrt{2\pi }}$	[11]

**Table 4:** Parameters used in the model equations listed in Table 3.

Symbol	Parameter	Value	Reference
α	Tumour dose-proportional radiosensitivity coefficient	Sampled from lognormal population distribution	N/A
α¯	Mean value of α distribution	0.15 Gy^−1^	[12]
$\sigma_{\alpha }$	Standard deviation of α distribution	0.04 Gy^−1^	[12]
α/β	Fractionation-correction parameter for tumour or OAR	3.1 Gy (tumour) 5.4 Gy (rectum) 8.0 Gy (bladder)	[12] [16] [17,18]
ρ	Tumour clonogen density (voxel-specific)	Variable (population median 10^7^ cells per whole prostate volume)	[12]
V	Voxel volume (resolution-dependent)	2 × 2 × 2.5 mm^3^	N/A
n	Number of treatment fractions	5	N/A
d	Dose per fraction per voxel	Variable	N/A
$T_{exp}$	Overall treatment time	Approximated as 2n days	N/A
$T_{pot}$	Potential tumour doubling time	42 days	[12]
M	Number of samples from population α distribution	36	N/A
$w\left(\alpha_{k}\right)$	Weight factors normalising the α distribution such that $\sum_{k\;=1}^{M}w\left(\alpha_{k}\right)=1$	Variable	N/A
OER	Oxygen enhancement ratio	1.4 (1.2–1.8)	[12]
N	Number of voxels comprising structure (prostate or OAR)	Variable	N/A
$\Sigma^{\prime }$	Effective systematic geometric error	Variable	[19,20,21]
$G_{\Sigma^{\prime }}$	Gaussian distribution of effective systematic error	Variable	N/A
a	EUD model scaling parameter	See Table 5	N/A
TD50	OAR uniform dose which will lead to complications in 50% of the population	See Table 5	N/A
m	NTCP model slope	See Table 5	N/A

**Table 5:** Dose constraints used in treatment planning optimisation.

Planning Method	VOI	Constraints
(1) Uniform-dose	CTV	V35Gy ≥ 99%
	PTV	V33.25Gy ≥ 99%
	Rectum	V28Gy ≤ 15% V32Gy ≤ 20%
	Bladder	V28Gy ≤ 15% V32Gy ≤ 20%
	HOF	V28Gy ≤ 5%
(2) Focal DE to tumour *	GTV	V35Gy ≥ 99%; aimed up to 50 Gy ^⁑^ V52Gy ≤ 0.1cc
(3) Focal DE to tumour and hypoxic volume *	GTV	V35Gy ≥ 99%; aimed up to 50 Gy ^⁑^
	HTV	V35Gy ≥ 99%; aimed up to 60 Gy ^⁑^ V62Gy ≤ 0.1cc
(4) Biological optimisation	CTV	Maximise <TCP> V35Gy≥ 99%
	Rectum	Minimise <NTCP> TD50 = 78.4 Gy, m = 0.108, a = 6
	Bladder	Minimise <NTCP> TD50 = 80 Gy, m = 0.11, a = 6
	HOF	V28Gy ≤ 5%

* = OAR constraints identical to uniform-dose planning, ⁑ = doses up to prescription as long as OAR constraints are met.

**Table 6:** The overall distribution of geometric uncertainties considered in dose-based planning methods (Methods 1–3), and those considered in biological optimisation (Method 4). $\Sigma^{\prime }$ = effective systematic error.

Uncertainties (mm)	Dose-Based Planning			Biological Optimisation
	Mean	$\Sigma^{\prime }$	Margin	$\Sigma^{\prime }$
AP	−0.86	3.6	9.0	5.3
LR	−0.03	2.6	6.5	4.6
SI	0.21	3.2	8.0	5.0

## Data Availability

The data presented in this study are available on request from the corresponding author. The data are not publicly available due to privacy restrictions.
